# Genotyping by sequencing resolves shallow population structure to inform conservation of Chinook salmon (*Oncorhynchus tshawytscha*)

**DOI:** 10.1111/eva.12128

**Published:** 2014-01-02

**Authors:** Wesley A Larson, Lisa W Seeb, Meredith V Everett, Ryan K Waples, William D Templin, James E Seeb

**Affiliations:** 1 School of Aquatic and Fishery Sciences, University of WashingtonSeattle, WA, USA; 2 Gene Conservation Laboratory, Alaska Department of Fish and GameAnchorage, AK, USA

**Keywords:** Chinook salmon, effective population size, genetic stock identification, population genomics, RAD sequencing, SNPs, western Alaska

## Abstract

Recent advances in population genomics have made it possible to detect previously unidentified structure, obtain more accurate estimates of demographic parameters, and explore adaptive divergence, potentially revolutionizing the way genetic data are used to manage wild populations. Here, we identified 10 944 single-nucleotide polymorphisms using restriction-site-associated DNA (RAD) sequencing to explore population structure, demography, and adaptive divergence in five populations of Chinook salmon (*Oncorhynchus tshawytscha*) from western Alaska. Patterns of population structure were similar to those of past studies, but our ability to assign individuals back to their region of origin was greatly improved (>90% accuracy for all populations). We also calculated effective size with and without removing physically linked loci identified from a linkage map, a novel method for nonmodel organisms. Estimates of effective size were generally above 1000 and were biased downward when physically linked loci were not removed. Outlier tests based on genetic differentiation identified 733 loci and three genomic regions under putative selection. These markers and genomic regions are excellent candidates for future research and can be used to create high-resolution panels for genetic monitoring and population assignment. This work demonstrates the utility of genomic data to inform conservation in highly exploited species with shallow population structure.

## Introduction

Discrete management of genetically distinct populations can increase species-wide resilience and stabilize the productivity of ecosystems as a whole (Hilborn et al. 2003; Schindler et al. 2010). For over three decades, genetic data from 10 to 100 putatively neutral markers have been used to identify discrete populations, define conservation units, and estimate demographic parameters (Utter et al. 1974; Wirgin and Waldman 1994; Waples et al. 2008). The use of genetic data for management has been especially successful in Pacific salmon (*Oncorhynchus spp*.) which exhibit extensive population structure (Utter and Ryman 1993; Shaklee et al. 1999). However, applications have been limited for recently isolated populations of salmonids (Taylor et al. 1997) or marine species with little neutral structure (Waples 1998). In these circumstances, data from thousands of markers (genomic data) may be necessary to resolve population structure and aid management.

Genomic data can provide accurate estimates of neutral population structure (Avise 2010; Funk et al. 2012; Narum et al. 2013), identify genomic regions that display adaptive divergence (Allendorf et al. 2010; Angeloni et al. 2012), and provide increased accuracy when estimating demographic parameters (Allendorf et al. 2010). Genotypes from thousands of loci have been used to elucidate neutral structure in populations of Pacific lamprey (*Entosphenus tridentatus*, Hess et al. 2013) and to improve resolution of fine-scale structure in Atlantic salmon (*Salmo salar*, Bourret et al. 2013). Additionally, genome scans have revealed adaptively important markers and genomic regions in sockeye salmon (*Oncorhynchus nerka*, Russello et al. 2012), Atlantic cod (*Gadus morhua*, Bradbury et al. 2013; Hemmer-Hansen et al. 2013), and lake whitefish (*Coregonus clupeaformis*, Renaut et al. 2012). Although many studies have used genomic data to elucidate structure in nonmodel organisms, demographic parameters such as effective size are rarely estimated with these types of data.

Effective population size (*N*_e_) is an important parameter in conservation biology (Frankham 2005), but methods to calculate *N*_e_ with genomic data are lacking (Waples and Do 2010). Specifically, many calculations of *N*_e_ require knowledge of linkage relationships, which are often unknown for nonmodel organisms. A possible solution to this problem is the use of high-density linkage maps that can now be created rapidly for many species with genotyping by sequencing (e.g., Baxter et al. 2011; Miller et al. 2012; Gagnaire et al. 2013). Using data from these maps, it is possible to obtain estimates of *N*_e_ that are not biased by physical linkage. To the best of our knowledge, this method has only been implemented in populations of model organisms (Park 2011; Sved et al. 2013), but the increasing availability of linkage maps will facilitate *N*_e_ estimation in many species of conservation concern.

**Figure 1:**
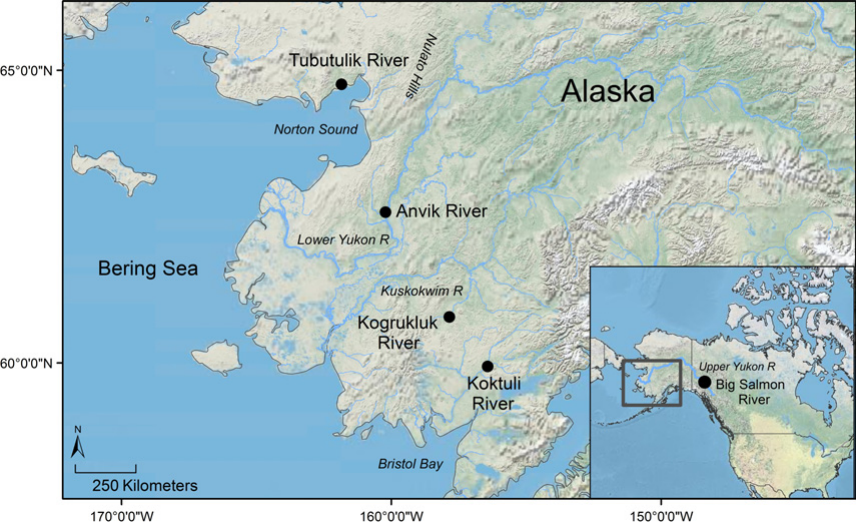
Map of sampling locations. See Table 1 for additional details about each sampling site.

Chinook salmon (*Oncorhynchus tshawytscha)* from western Alaska represent an excellent system to explore the utility of genomics in a management context. Chinook salmon inhabit four major regions in western Alaska: Norton Sound, the Yukon River, the Kuskokwim River, and Bristol Bay, all of which vary significantly in size, hydrology, and climate (Fig. 1, Olsen et al. 2011). The Kuskokwim and Yukon regions are composed of a mainstem river with many tributaries, whereas Norton Sound is composed of many unconnected and short rivers (mean length ∼110 km; Olsen et al. 2011). The Bristol Bay region is composed of several river systems each with smaller tributaries (i.e., Nushagak, Togiak, Naknek rivers). Past studies using allozymes and single-nucleotide polymorphisms (SNPs) found evidence of structure in western Alaska, but concluded that differences among populations in Norton Sound, the lower portions of the Yukon and Kuskokwim rivers, and Bristol Bay, were insufficient to allocate mixture samples back to their region of origin (Gharrett et al. 1987; Templin et al. 2011). Returns of Chinook salmon to western Alaska over the past decade have been approximately 20% lower than their long-term average, renewing interest in the migration patterns and vulnerability of stocks to fisheries in this region (ADF&G 2013). Improved resolution of population structure would allow managers to investigate these questions using genetic tools. Additionally, estimates of *N*_e_ and other demographic parameters could help to inform conservation and management efforts across the region.

We used restriction-site-associated DNA (RAD) sequencing to investigate the population structure and demography of Chinook salmon from western Alaska. We identified over 10 000 SNPs in 270 individuals from five populations across western Alaska. Patterns of genetic variation were assessed using both population-and individual-based methods and validated with assignment tests. We then aligned our RAD markers to a linkage map to calculate *N*_e_ with and without removing physical linkage. We also conducted outlier tests and used the linkage map to detect loci and genomic regions under putative selection. This approach defines an important way that genomics can be used to inform management of nonmodel species with high gene flow.

## Materials and methods

### Tissue sampling

Tissue samples from spawning Chinook salmon were available from four regions in coastal western Alaska and one in the upper Yukon River (Fig. 1, Table 1). We selected populations that did not have unusually small census sizes and that were genetically similar to proximate populations identified from previous studies (Olsen et al. 2011; Templin et al. 2011); this approach ensured that our conclusions were based on populations that were representative of each region. Chinook salmon from the upper Yukon River are highly differentiated from those of western Alaska (Smith et al. 2005; Templin et al. 2011) and were included to anchor inferences of population structure.

**Table 1:** Populations analyzed in this study with year sampled, sample size (*N*), observed heterozygosity (*H*_O_), and expected heterozygosity (*H*_E_).

Sampling location	Region	Year	GPS coordinates	*N*	*H*_O_	*H*_E_
Tubutulik River	Norton Sound	2009	64.740, −161.888	56	0.248	0.252
Anvik River	Lower Yukon R	2007	62.681, −160.214	54	0.260	0.261
Kogrukluk River	Kuskokwim R	2007	60.841, −157.846	57	0.251	0.258
Koktuli River	Bristol Bay	2010	59.935, −156.427	56	0.256	0.259
Big Salmon River	Upper Yukon R	2007	61.867, −134.917	47	0.232	0.232

### RAD sequencing, SNP discovery and genotyping

Restriction-site-associated DNA libraries were prepared with the restriction enzyme *SbfI* following the methods of Baird et al. (2008) and Everett et al. (2012) and sequenced on an Illumina HiSeq2000 at the University of Oregon Genomics Core Facility. We constructed 18 libraries for single-end sequencing (100 bp target length) containing 12–24 individuals per library and one library for paired-end sequencing (100 × 2 bp target) containing eight individuals to assemble longer sequence contigs for annotation. Pooled individuals were identified with unique 6-bp barcodes.

We used the *Stacks* software package, version 0.9999 (Catchen et al. 2011) and methods similar to Hohenlohe et al. (2013) to discover and genotype SNPs from the sequenced RAD tags. Quality filtering of raw reads and demultiplexing based on barcode was conducted using *process_radtags*. Stacks of similar sequences were then assembled in each individual with *ustacks*, and a catalog of loci was created with *cstacks*. We included only the two individuals from each population with the greatest amount of sequence data when creating our catalog to reduce the detection of false polymorphisms. Including more individuals per population would have facilitated the detection of low-frequency SNPs, but would not have added additional SNPs to the final data set because these low-frequency SNPs were filtered out in downstream analyses. Finally, we used *sstacks* and *populations* to combine the genotypes from each individual into a single *Genepop* formatted file.

### SNP validation

Putative SNPs discovered using *Stacks* were filtered to remove possible sequencing errors, paralogous sequence variants (PSVs), and uninformative polymorphisms. First, we removed any putative SNP that failed to genotype in >80% of individuals. We then removed those with a minor allele frequency <0.05 in all populations. These polymorphisms are likely to be uninformative, are difficult to distinguish from sequencing errors, can distort signals of selection and drift in natural populations, and may bias tests for selection (Roesti et al. 2012). We also discarded putative SNPs that were found at RAD tag positions >87 bp because these positions contained more polymorphisms on average than the rest of the sequence (138 per bp for bp 1–87, 223 per bp for bp 88–93). This increase in putative SNPs per base pair is likely a result of sequencing errors as Illumina sequencing is more error prone toward the terminal positions of reads (Minoche et al. 2011). We kept only the putative SNP with the highest *F*_ST_ from each RAD tag to reduce linkage in our data set. We also used the program *PLINK* version 1.07 (Purcell et al. 2007) to test for linkage disequilibrium between each pair of loci. If a locus pair had an *r*^2^ value >0.8 in three of five populations, we removed the locus that was genotyped in the fewest individuals.

Paralogous sequence variants, which are abundant in salmonids as a result of an ancient whole-genome duplication event (Allendorf and Thorgaard 1984; Seeb et al. 2011), were removed from the data set when possible. PSVs are closely related sequences from different genomic locations that do not segregate as single loci and are therefore difficult to genotype accurately (Gidskehaug et al. 2011). Haploid individuals can be used to identify PSVs because PSVs will appear heterozygous when all correctly segregating loci are homozygous (Hecht et al. 2013). To screen for PSVs in our data, we genotyped 50 haploid Chinook salmon from Washington, USA, at all putative SNPs discovered above, and loci with >10% heterozygosity were removed.

We also conducted exact tests of Hardy–Weinberg equilibrium in *Genepop* version 4 (Rousset 2008) and removed loci that were out of equilibrium in three or more populations (*P *<* *0.05). We then removed individuals that were missing genotypes at > 15% of the SNPs. As a final filtration step, we used *ML-Relate* (Kalinowski et al. 2006) to look for duplicated individuals in our data.

### Paired-end assembly and *BLAST* annotation

We conducted a paired-end assembly with the P1 and P2 reads from each locus using *Velvet* (v.1.1.06, Zerbino and Birney 2008) and the methods of Etter et al. (2011) and Everett et al. (2012) to increase query lengths for *BLAST* annotation. Consensus sequences were then aligned to the Swiss-Prot database using the *BLASTX* search algorithm. Alignments with E-values of ≤10^−4^ were retained. If multiple alignments had E-values of ≤10^−4^ for the same locus, the alignment with the lowest *E*-value was retained.

### Population structure and assignment tests

**Figure 2:**
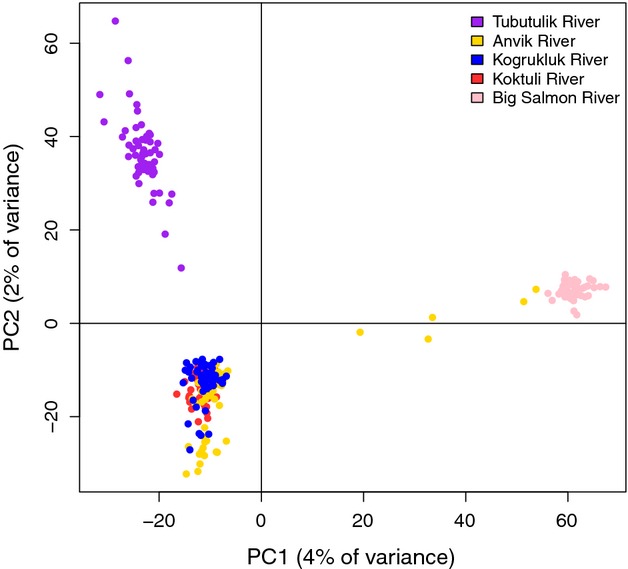
Individual-based principal component analysis for all populations and 10 944 SNPs. The five intermediate individuals from the Anvik River were removed from further analyses (see).

Initial analysis of population structure was conducted with an individual-based principal component analysis (PCA) implemented in the R package *adegenet* (Jombart 2008). The significance of each principal component was assessed by randomly permuting the data 1000 times and comparing the observed eigenvalues to values generated by conducting PCA on the permuted data. PCA revealed five nonconforming individuals in the Anvik River collection that grouped between the Big Salmon River and Anvik River clusters (Fig. 2). These five individuals were removed from further analyses as they likely represent transient fish from middle or upper Yukon River populations. After removing these individuals, we calculated pairwise *F*_ST_ values (Weir and Cockerham 1984) for each population and performed significance tests for genetic differentiation in *Arlequin* 3.5 (Excoffier and Lischer 2010) using an exact test with 10 000 permutations.

We conducted an analysis of molecular variance (amova) in *Arlequin* 3.5 to examine the variation within and among groups of genetically similar populations. The hierarchy for this analysis was chosen based on the clustering from the PCA: (i) Koktuli River, Kogrukluk River, and Anvik River, (ii) Tubutulik River, and (iii) Big Salmon River. Separate amovas were conducted for (i) the entire data set and (ii) all populations except the Big Salmon River. Finally, we calculated global and per-locus observed and expected heterozygosities for each population in *GenAlEx* 6.5 (Peakall and Smouse 2012).

We examined fine-scale structure in the closely related Anvik River, Kogrukluk River, and Koktuli River populations with an individual PCA including only these three populations (see above for PCA methods). This analysis was conducted separately for the 10 944 RAD SNPs and 39 of the 43 SNPs from Templin et al. (2011) that were developed for Chinook salmon from expressed sequence tags. Of the four SNPs from Templin et al. (2011) that were not genotyped, two were removed because they were essentially monomorphic in other populations from western Alaska and two were removed because they were in linkage disequilibrium with another locus (Templin et al. 2011).

Assignment power of four panels was evaluated with leave-one-out tests in *GeneClass2.0* (Piry et al. 2004) to compare the influence of number of SNPs and relative divergence of SNPs on assignment accuracies. The four panels were (i) 39 SNPs from Templin et al. (2011), (ii) 39 randomly chosen SNPs from the complete data set of 10 944 RAD SNPs, (iii) the complete data set of 10 944 RAD SNPs, and (iv) the full set of RAD SNPs with the 733 outlier SNPs that were found to be under putative selection removed. We did not construct a panel with only the most divergent RAD SNPs because this approach would have led to an upward bias in the predicated accuracy of assignment for that panel (Anderson 2010). Leave-one-out tests were conducted by removing an individual from the baseline without replacement then assigning that individual back to a reference population using a Bayesian approach described in Rannala and Mountain (1997). Individuals were considered to be assigned to a population if the assignment probability to that population was higher than to any other population.

### Alignment to linkage map

We aligned our filtered loci to a linkage map for Chinook salmon consisting of 3534 RAD-derived SNPs distributed across 34 linkage groups ranging in size from 27.75 to 160.23 cM (Table S1, Everett and Seeb 2014). To conduct the alignments, we used *BLASTN* (Altschul et al. 1990) with the following parameters: minimum alignment length of 90 bp, 95% identity, and no more than two mismatching bases. If a single locus aligned to multiple map loci, we discarded all alignments for that locus. We used relatively strict alignment parameters for this analysis because sequence alignment in tetraploid-origin salmonids can provide ambiguous results when alignment parameters are not sufficiently strict (Everett et al. 2011; Seeb et al. 2011).

### Calculating *N*_e_ and *N*_e_/*N*

Estimates of *N*_e_ were performed with the linkage disequilibrium method (Hill 1981; Waples 2006) updated for missing data following Peel et al. (2013). This method assumes all loci in the analysis are physically unlinked then utilizes the observed linkage disequilibrium to estimate *N*_e_. We removed comparisons between loci on the same linkage groups to obtain estimates that were unbiased by physical linkage (Park 2011; Sved et al. 2013). Additionally, we removed all loci that were putatively under selection as suggested by Waples (2006) (see below for description of tests for loci under selection). Calculations of *N*_e_ were conducted using *N*_*e*_*Estimator* (Do et al. 2014) and *R* (R core development team 2011). *N*_*e*_*Estimator* was used to calculate *r*^2^ values for each locus pair with the following parameters: a minimum allele frequency cutoff of 0.02 and a random mating model. We then implemented the methods described in Waples (2006) and Peel et al. (2013) in *R* to obtain *N*_e_ estimates and parametric 95% confidence intervals for each population (scripts available from W. Larson upon request). We calculated *N*_e_ for three data sets: (i) all RAD SNPs that aligned to the linkage map, (ii) all RAD SNPs that aligned to the map with pairwise comparisons between markers on the same linkage group removed, and (iii) the 39 SNPs from Templin et al. (2011) that were in linkage equilibrium.

We calculated the ratio of effective size to census size (*N*_e_/*N*) using *N*_e_ calculated with RAD-derived SNPs after removing physical linkage and estimates of total escapement obtained from aerial surveys (Koktuli River, Anvik River, Tubutulik River) and weir counts (Kogrukluk River, Big Salmon River). Multiple aerial surveys were used to estimate total run size for the Anvik River and Koktuli River populations, but only single aerial counts were available for the Tubutulik River population. Single aerial counts from a river near the Tubutulik River collection were approximately four times smaller than those taken from a counting tower; therefore, we multiplied the aerial count from our collection by four. We averaged the last ten years of data to obtain an approximate value of census size for each population (only last 3 years used for Koktuli River due to data availability).

Estimates of *N*_e_ for Chinook salmon populations are complicated by the fact that multiple cohorts are represented in each spawning group (Waples 1990). Single-sample estimates of *N*_e_ therefore do not precisely reflect the effective number of breeders per year or the effective number of breeders per generation, but instead represent some intermediate value. We calculated two *N*_e_/*N* ratios to bracket these possible scenarios: *N*_e_ divided by the average census size (escapement) per year (*N*_e_/*N*) and *N*_e_ divided by the total census size per generation (*N*_e_/NG). Values of G for each population were obtained from the sources in Table 5 by averaging age compositions across one to 38 years of data depending on availability.

### Detection of loci under putative selection

We identified putative loci under selection with *Arlequin* 3.5. This program uses coalescent simulations to create a null distribution of F-statistics then generates *P*-values for each locus based on this distribution and observed heterozygosities across loci (Excoffier et al. 2009). A hierarchical island model was selected to reduce false positives introduced due to underlying population structure (Excoffier et al. 2009). The population hierarchy was the same as in the amova. Settings for the analysis were 20 000 simulations, 10 simulated groups, and 100 demes per group. Loci that fell above the 95% quantile of the *F*_ST_ distribution were considered candidates for directional selection.

### Detection of candidate genomic regions under selection

We used a linkage map in conjunction with a sliding window analysis to identify highly divergent regions of the genome that may be under selection (c.f., Bourret et al. 2013). This analysis was conducted with a sliding window approach that compares the mean pairwise *F*_ST_ of a small (5 cM) genomic region to a null distribution created by bootstrapping over the complete data set (Hohenlohe et al. 2010; Bourret et al. 2013). For each window, we sampled *N F*_ST_ values with replacement from the entire data set where *N* was the number of SNPs in the window. This resampling routine was repeated 1000 times to generate a null distribution. Windows with mean *F*_ST_ values above the 95% quantile of the null distribution were candidates for directional selection. If a window mean was above 90% after 1000 replicates, we increased the number of replicates to 5000 to improve accuracy in the tails of the null distribution. We chose a sliding window size of 5 cM and frame shift value of 1 cM. We also required at least two SNPs to be present in a window to conduct the above test. After testing multiple window sizes, we found that a 5-cM window provided sufficient resolution for detecting divergent regions without introducing excessive variance. This value was also used by Bourret et al. (2013) for linkage groups with similar numbers of markers to ours. We conducted this analysis for all pairwise population comparisons.

## Results

### Sequencing, SNP discovery and filtration

We obtained RAD data from 289 individuals, and the number of sequences obtained for each individual ranged from 1 622 400 to 8 707 337 with an average of 3 796 368 (excluding low-quality individuals, see below). Alignments using *Stacks* revealed 42 351 putative SNPs. Removing putative SNPs that were genotyped in <80% of individuals eliminated more than half of these, leaving 20 296. After removing polymorphisms in bp 87–94 of each RAD tag, removing all but one putative SNP from each tag, and removing SNPs with minor allele frequency <0.05, 12 585 SNPs remained. Screens for paralogous sequence variants revealed 845 loci that were potentially duplicated; these loci were eliminated. Significant deviations from Hardy–Weinberg equilibrium were observed in 397 SNPs, and these loci were also removed. Significant linkage disequilibrium in three or more populations was found for 399 SNPs, and one SNP from each pair was removed. The final filtered data set consisted of 10 944 SNPs. We removed 17 individuals that were genotyped in <85% of SNPs, seven from the Kogrukluk River, four from the Anvik River, and six from the Big Salmon River (adjusted sample sizes in Table 1). Relatedness analysis revealed two pairs of duplicated individuals (R > 0.9) from the Anvik River population, and one individual from each pair was removed. The final filtered data set consisted of 270 individuals genotyped at 10 944 SNPs. Summary statistics for each locus are available in Table S1, and histograms of overall and pairwise *F*_ST_ for each locus are in Fig. S1.

### Paired-end assembly and *BLAST* annotation

Paired-end assemblies produced 11 666 contigs with an average length of 268 bp (minimum 150 bp, maximum 565 bp). *BLAST* annotation of these contigs yielded significant hits for 1576 (14%) of 10 944 SNPs (Table S2). Of these hits, over one-third aligned to transposable elements. Other common functional groups included DNA polymerases and transmembrane proteins.

### Population structure

Principal component analysis revealed that the Big Salmon River and Tubutulik River populations formed completely separate clusters, while the Koktuli River, Kogrukluk River, and Anvik River populations essentially formed a single cluster (Fig. 2). The overall *F*_ST_ of the full data set was 0.041, and pairwise *F*_ST_ values ranged from 0.003 for the Koktuli River–Kogrukluk River comparison to 0.098 for the Big Salmon River–Tubutulik River comparison (Table 2). Genetic differentiation between all population comparisons was highly significant (*P *<* *0.001). The results of these significance tests should, however, be interpreted with extreme caution due to the large number of loci, which may overestimate precision.

**Table 2:** Pairwise *F*_ST_ values calculated using 10 944 SNPs and number of genomic regions that were under putative selection (in parentheses). All pairwise comparisons are significantly differentiated (*P *< 0.01).

	Tubutulik River	Anvik River	Kogrukluk River	Koktuli River
Anvik River	0.030 (20)			
Kogrukluk River	0.027 (20)	0.005 (20)		
Koktuli River	0.028 (20)	0.006 (23)	0.003 (20)	
Big Salmon River	0.098 (24)	0.075 (25)	0.075 (21)	0.077 (25)

We conducted hierarchical amova for the entire data set and for a data set without the Big Salmon River population (Table 3). Both analyses displayed much larger variation among groups than within groups. Levels of observed heterozygosity across populations ranged from 0.232 for the Big Salmon River to 0.260 for the Anvik River (Table 1).

**Table 3:** Results from two amovas with 10 944 SNPs.

Source of variation	d.f.	Percentage of variation
*All populations*		
Among groups	2	5.26
Among populations within groups	2	0.45
Within populations	529	94.32
*Big Salmon River excluded*		
Among groups	1	2.41
Among populations within groups	2	0.43
Within populations	436	97.18

**Figure 3:**
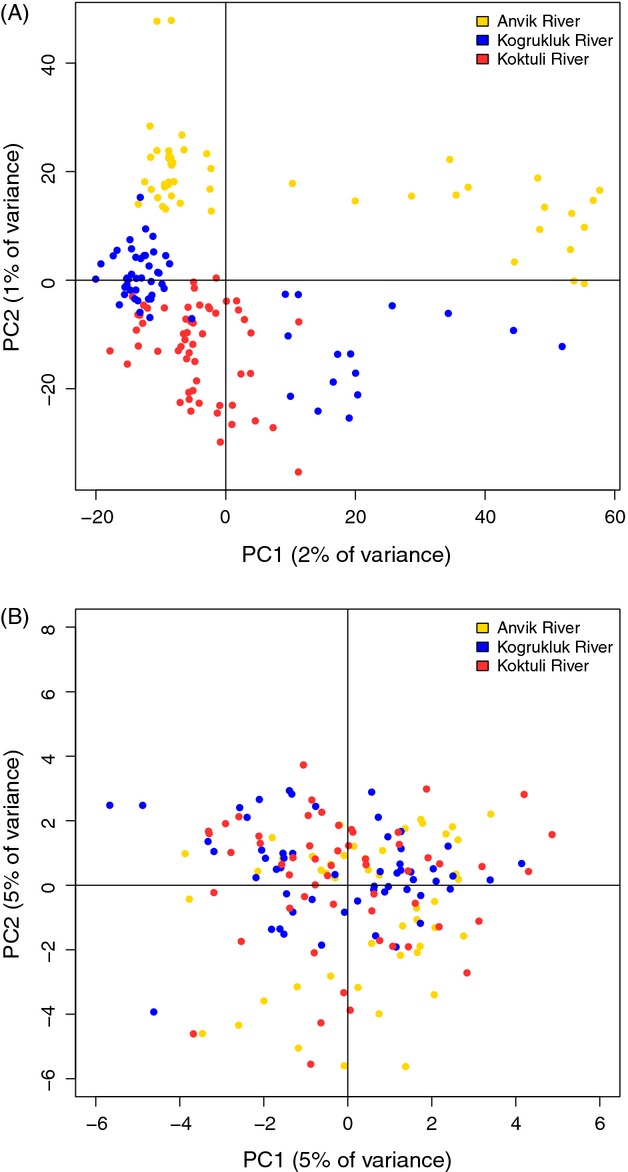
Individual-based principal component analysis for the Anvik River, Kogrukluk River, and Koktuli River populations using (A) 10 944 RAD SNPs and (B) 39 SNPs from Templin et al. (2011).

When the Koktuli River, Kogrukluk River, and Anvik River populations were analyzed separately with 10 944 SNPs, all populations generally formed discrete clusters, but some overlap was present between the Koktuli River and Kogrukluk River populations (Fig. 3A). Additionally, populations from the Anvik River and Kogrukluk River each contained a subset of 10–20 individuals that fell outside the main cluster. When PCA was conducted with the 41 SNPs from Templin et al. (2011), no clustering pattern was apparent (Fig. 3B).

The relatively small amount of variation (1–5%) explained by the first and second principal components (PCs) in our PCAs (Figs 2 and 3) can be attributed to the large number of axes used. Each PCA contained as many axes as individuals plotted, so PCA using all populations contained 270 axes, and the PCA with three populations contained 163. PCs one and two in both PCAs each explained more than three times the variation of the average axis and explained significantly more variation than would be expected if no real correlation existed (*P* < 0.001), but because of the large number of axes, the actual proportion of variation explained was small.

Assignment accuracy was much higher using >10 000 SNPs (≥ 89% assignment to correct population) compared to 39 SNPs (∼50% assignment to correct population, Table 4). Panels containing close to the same number of SNPs generally performed similarly, but the 39 SNPs from Templin et al. (2011) did perform slightly better than the 39 randomly chosen RAD SNPs, and the panel containing all 10 944 RAD SNPs performed slightly better than the panel with the 733 outlier SNPs removed (Table 4).

**Table 4:** Results of leave-one-out tests for individual assignment with four SNP panels. Panels are: (1) 39 EST: 39 SNPs previously developed for Chinook salmon from expressed sequence tags (ESTs, Templin et al. 2011), (2) 39 RAD: 39 randomly chosen SNPs from the complete data set of 10 944 RAD SNPs, (3) 10 944 RAD: the complete data set of RAD SNPs, and (4) 10 211 RAD no outliers: the full set of RAD SNPs with the 733 outlier SNPs that were found to be under putative selection removed. Individuals were considered to be correctly assigned if the assignment probability to population of origin was higher than to any other population. See Table S3 for assignment probabilities for each individual.

Regions	% Correct assignment			
	39 EST	39 RAD	10 944 RAD	10 211 RAD no outliers
Tubutulik River	67	65	100	100
Anvik River	46	30	91	89
Kogrukluk River	34	30	93	93
Koktuli River	29	30	98	95
Big Salmon River	96	87	100	100

### Alignment to linkage map

Of the 10 944 filtered loci, 1156 were successfully placed on the linkage map (33% of loci on the map successfully aligned to one of the 10 944 loci discovered in populations from western Alaska, see Table S1 for map location of successful alignments). This proportion may seem small, but it is important to note that the map was constructed using a single Chinook salmon from Washington State. Chinook salmon from Washington State are substantially diverged from populations in western Alaska (Templin et al. 2011), therefore, it is likely that many RAD tags did not contain loci that were polymorphic in both the mapping cross and our study populations and were not useful for our analyses. Additionally, because only one individual was used for the mapping cross, our alignments were limited to the RAD tags containing SNPs that segregated in the mapped individual.

### Demographic estimates

Estimates of *N*_e_ with the RAD-derived SNPs were highly variable across populations, ranging from close to 500 in the Anvik River to infinity for the Koktuli River (Table 5). These estimates were calculated using SNPs that were successfully aligned to the linkage map, providing over 500 000 pairwise comparisons between loci. Pairwise comparisons between SNPs located on the same linkage group represented about 20 000 of the 500 000 comparisons (6%). These 20 000 comparisons were removed to estimate *N*_e_ between physically unlinked loci. Estimates of *N*_e_ were consistently smaller for the data set that included all comparisons (Table 5). This downward bias was not uniform, however, as estimates from Norton Sound appeared to be more affected by linkage than estimates for the other populations.

**Table 5:** Estimates of effective population size (*N*_e_) for five populations calculated with 1118 RAD-derived SNPs that were placed on the linkage map and 39 of the 43 SNPs that were in linkage equilibrium from Templin et al. (2011). Estimates with RAD SNPs are calculated using only comparisons between loci on different linkage groups (*N*_e_ linkage removed) and all comparisons (*N*_e_ all data). The ratio of effective population size to census size (*N*_e_/*N*) and effective population size to census size multiplied by generation length (*N*_e_/NG) for each population is also reported (G is generation length and *N* is an approximate value of yearly escapement for each population, see methods). The *N*_e_ used for these calculations is *N*_e_ linkage removed (column 2). We did not calculate *N*_e_/*N* or *N*_e_/NG for the Bristol Bay and upper Yukon populations because confidence intervals included infinity, suggesting our point estimates may not be completely representative.

Population	*N*_e_ linkage removed	*N*_e_ all data	*N*_e_ 39 SNPs	G	*N*	*N*_e_/*N*	*N*_e_/NG	Source of *N*	Source of G
Tubutulik River	1909 (1295–3602)	808 (674–1009)	Inf (174–Inf)	5.43	3100	0.62	0.11	Banducci et al. (2007)	Lingnau (1996)
Anvik River	516 (451–604)	505 (443–586)	209 (65-Inf)	5.48	1700	0.30	0.06	Howard et al. (2009)	Sandone (1995)
Kogrukluk River	2026 (1375–3825)	1723 (1233–2842)	Inf (134–Inf)	5.20	12 000	0.17	0.03	Williams and Shelden (2011)	Howard et al. (2009)
Koktuli River	Inf (6055-Inf)	26 071 (3733-Inf)	Inf (Inf-Inf)	5.13	6000	N/A	N/A	Woody (2012)	Howard et al. (2009)
Big Salmon River	13 101 (1505-Inf)	4243 (1806-Inf)	520 (70-Inf)	5.65	5000	N/A	N/A	Mercer and Wilson (2011)	Howard et al. (2009)

Estimates of *N*_e_ with the 39 SNPs from Templin et al. (2011) ranged from 209 for the Anvik River to infinity for the Koktuli River, Kogrukluk River, and Tubutulik River populations. Confidence intervals for each estimate using 39 SNPs included infinity and were larger than confidence intervals around estimates from the RAD-derived SNPs.

Estimates of *N*_e_/*N* and *N*_e_/NG were extremely variable, ranging from 0.17 and 0.03 for the Kogrukluk River population to 0.59 and 0.11 for the Tubutulik River population (Table 5). We did not calculate *N*_e_/*N* or *N*_e_/NG for the Koktuli River or Big Salmon River populations because the confidence intervals around *N*_e_ included infinity, suggesting our point estimates of *N*_e_ may not be completely representative.

### Loci and genomic regions under putative selection

Outlier tests in *Arlequin* revealed 733 loci (6.7%) that were significant outliers at the 5% level and 178 (1.6%) that were significant at the 1% level. *BLAST* annotation of the outliers at the 5% level revealed 96 significant hits (13% success rate). Transposable elements represented over one-third of the significant hits which is consistent with the pattern from the complete data set.

**Figure 4:**
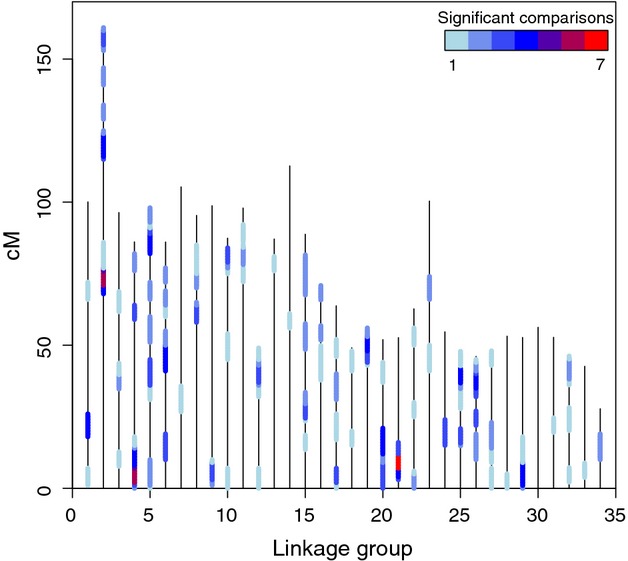
Regions of the genome under putative selection as inferred by pairwise *F*_ST_ across all population pairs. Each vertical line represents a linkage group, and the length of the line is proportional to the size of the linkage group in cM. Shaded areas indicate regions which are significantly diverged in at least one population pair indicating putative selection. The color of the shading corresponds to the number of significant pairwise population comparisons with red and purple indicating over half of the population pairs are divergent in the given region.

The number of genomic regions under putative selection for each population pair ranged from 20 to 25 and generally increased when the Big Salmon River population was included (Fig. 4, Table 2). Overall, these regions appeared to be scattered randomly throughout the genome and were often significant in only one or two population comparisons. Despite this pattern, three genomic regions on separate linkage groups (LG) were candidates for selection in more than half of the population comparisons (Fig. 4). These regions are LG2 at 70–78 cM, LG4 at 2–8 cM, and LG21 at 7–12 cM.

## Discussion

We used RAD sequencing to characterize the genetic structure, genomic divergence, and demography of five populations of Chinook salmon from western Alaska. Patterns of genetic differentiation were similar to but more identifiable than in past studies (Gharrett et al. 1987; Templin et al. 2011). Estimates of population *N*_e_ ranged from 516 to infinity and appeared to be biased downward when loci that were physically linked were not removed. Regions of putative adaptive divergence appeared to be randomly distributed across the genome with few shared areas of high divergence across populations, but we did find three genomic regions that displayed high divergence in multiple populations. Using genomic data, we were able to conduct individual assignment in populations where it was previously unfeasible, discover genomic regions under putative selection, and estimate *N*_e_ in populations with >1000 individuals. Our approach therefore represents a significant improvement over previous studies employing fewer markers and no linkage map.

### Population structure

The largest genetic differentiation between populations in our data set existed between the Big Salmon River from the upper Yukon River and all other coastal populations. Chinook salmon from the upper Yukon River are thought to have genetically diverged from coastal populations after being isolated during the last glacial maximum (Olsen et al. 2011). Our results support this hypothesis and are consistent with those based on allozymes, microsatellites, and SNPs (Gharrett et al. 1987; Olsen et al. 2010; Templin et al. 2011).

We also found high levels of divergence between the Tubutulik River in Norton Sound and all other populations. This divergence was likely facilitated by the Nulato Hills, a small mountain range that separates the tributaries of Norton Sound from those of the Yukon River (Fig. 1), but could have also been influenced by environmental characteristics such as precipitation (Olsen et al. 2011).

Populations from the lower Yukon, Kuskokwim, and Bristol Bay regions (Anvik River, Kogrukluk River, and Koktuli River) were least divergent, displaying pairwise *F*_ST_ values < 0.01 for all population comparisons. The relatively small divergence we observed is consistent with other salmonids in the region (Olsen et al. 2011; Garvin et al. 2013) and is somewhat expected given the surrounding environment. Western Alaska is characterized by moisture-laden tundra and dynamic rivers that frequently change paths. When such stream captures occur, gene flow is facilitated between populations that were previously isolated. It is therefore likely that substantial historic and possibly continuing low-level gene flow has largely restricted genetic differentiation in this region (Seeb and Crane 1999).

Nevertheless, we found genetic structure among the Anvik River, Kogrukluk River, and Koktuli River populations using both individual-based clustering methods and assignment tests. The Anvik River population displayed the highest levels of divergence, forming a completely isolated cluster. This population may have diverged more quickly as a reflection of its relatively small estimated census and effective sizes (*N* = 1700, *N*_e_=516). The Kogrukluk River and Koktuli River populations, on the other hand, are at least four times larger than the Anvik River population and may not have been affected as substantially by genetic drift. Individuals that did not fall within major clusters were found in the Kogrukluk River and Anvik River populations. These individuals may represent evidence of gene flow from genetically diverged upriver populations, but could have also resulted from within population variation. Individual-based PCA using 39 SNPs from Templin et al. (2011) did not resolve the population structure that was observed with the RAD data and displayed no apparent clustering pattern. These results emphasize the utility of genome-wide data when attempting to elucidate patterns of population differentiation.

Assignment accuracies with both panels containing over 10 000 SNPs were ≥89% for all populations, while assignment accuracies with the panels containing 39 SNPs were close to 50% on average per population. Additionally, the inclusion of outlier loci only slightly improved assignment accuracy. These results indicate that a large number of neutral SNPs were sufficient to achieve precise assignment and that, for our analysis, the number of SNPs used seemed to have more influence on assignment accuracies than the resolution of those SNPs. Unfortunately, we were unable to evaluate the effectiveness of small panels of high-resolution SNPs compared to large panels of neutral SNPs because this type of analysis requires the use of a training and holdout data set (Anderson 2010), which was not feasible with the sample sizes in our study.

The patterns of population divergence observed here are consistent with previous studies, suggesting structuring of Chinook salmon populations on regional scales (Templin et al. 2011). Despite this pattern, sampling additional populations from each region would likely improve estimates of population divergence and assignment accuracy.

### Demography

Estimating and interpreting *N*_e_ in salmon populations using single samples can be difficult because multiple cohorts are often present (Waples 1990). *N*_e_ estimates therefore reflect a value somewhere between the effective number of breeders in a given year and the effective number of breeders per generation. We divided *N*_e_ by the census size (escapement) per year (*N*) and the census size per generation (NG) to account for both of these possibilities when comparing *N*_e_ to census size. The *N*_e_/*N* and *N*_e_/NG ratios were highly variable across our populations, indicating that effective and census size are not well correlated in our study system.

A meta-analysis of 251 estimates of *N*_e_/*N* found a median value of 0.14 and also showed that *N*_e_/*N* ratios are generally larger in smaller populations (Palstra and Ruzzante 2008). Larger *N*_e_/*N* ratios in smaller populations were also observed in our data. For example, the Anvik River had a census size of 1700 and *N*_e_/*N* of 0.30, while the Kogrukluk River had a census size of 12 000 and an *N*_e_/*N* of 0.17. This trend, however, was not consistent in the Tubutulik River population which had a census size of 3100 and *N*_e_/*N* ratio of 0.62.

The large *N*_e_/*N* ratio in the Tubutulik River population may have been due to gene flow from proximate populations which can introduce additional genetic diversity and inflate estimates of *N*_e_ (Palstra and Ruzzante 2011). The Tubutulik River is a small river in Norton Bay, which contains at least five additional salmon-producing rivers. Gene flow among subpopulations in this region may be quite common and could therefore have resulted in the larger than expected *N*_e_/*N* estimates that we observed. Gene flow from proximate populations may also be inflating *N*_e_ estimates from the Koktuli River and the Big Salmon River as both of these collections have census sizes close to 5000, but *N*_e_ estimates with confidence intervals including infinity. It is important to note that estimates of census size are approximate and may not be completely representative. Nevertheless, our results suggest that census size is not an adequate predictor of effective size, especially in populations that may belong to a larger metapopulation.

Removing comparisons between loci on the same linkage group appeared to have a nonuniform effect on estimates of *N*_e_ with larger estimates being more affected by removing linkage. For example, the estimate of *N*_e_ for the Anvik River population, the smallest population in the study, only changed by 10 when linked comparisons were removed, whereas the estimate for the Big Salmon River changed by almost 9000. This relatively small bias for small populations was also found by Sved et al. (2013) and is expected given that in small populations, the signal of linkage disequilibrium due to genetic drift should be large compared to the signal due to physical linkage.

It also appears that *N*_e_ estimates for populations of similar size can be affected nonuniformly by physically linked loci. Specifically, estimates of *N*_e_ for the Tubutulik River displayed a larger downward bias when physically linked loci were included than estimates for the Kogrukluk River, even though the effective sizes for these populations were similar with unlinked loci. The nonuniform effects we observed when removing physically linked loci may be due to historic signals of *N*_e_ that have been preserved due to linkage (Hill 1981; Tenesa et al. 2007).

Estimating *N*_e_ in large populations (*N*_e_ > 1000) with 10–100 genetic markers is extremely challenging due to the small amount of linkage disequilibrium caused by drift (Waples and Do 2010), but, with thousands of markers, accurately characterizing the signal of drift and estimating *N*_e_ may be feasible (Allendorf et al. 2010). Estimates of *N*_e_ from our study were infinite for three of five populations with 39 SNPs, but only infinite for one population with 1118 SNPs. Additionally, all estimates with 39 SNPs, but only two estimates with 1118 SNPs, displayed confidence intervals including infinity, and confidence intervals were consistently smaller with 1118 SNPs. Our results indicate that genomic data can improve the accuracy of *N*_e_ estimates in large populations, aiding management in many species.

### Putative adaptive divergence

We identified 6.7% of SNPs in our data set as outliers, consistent with past studies identifying 5–10% of markers as candidates for directional selection (Nosil et al. 2009). In general, patterns of divergence observed from our outliers were similar to patterns obtained using neutral markers. *BLAST* annotation of outlier loci revealed a high frequency of transposable elements, similar to the overall data set. These transposable elements are quite common in teleost fish and are generally assumed to behave as neutral markers (Radice et al. 1994) although some evidence suggests that they can be adaptively important (Casacuberta and González 2013).

Tests for genomic regions under putative selection revealed that these regions appeared to be spread randomly across the genome with few common ‘hot spots’ among populations. This pattern is consistent with Bourret et al. (2013), who found a similar distribution across the Atlantic salmon genome. Despite the apparent randomness, three regions were differentiated in more than five of ten population comparisons. These highly divergent regions may represent adaptively significant areas of the Chinook salmon genome and should be targets of future research. Population comparisons that included the Big Salmon River generally displayed the largest number of divergent regions. Although these regions likely represent adaptively significant areas of the genome, it is possible that at least a portion of them resulted from genetic drift as a result of isolation during the last glacial maximum (Olsen et al. 2011). Research aimed at disentangling signatures of drift from those of natural selection should therefore focus on systems with low neutral divergence across heterogeneous environments (Nielsen et al. 2009).

### Management and conservation implications for western Alaska

Returns of Chinook salmon to western Alaska have fallen dramatically over the last decade compared to their long-term average (ADF&G 2013). This precipitous decline has prompted multiple fisheries closures causing extensive economic hardship and threatening subsistence catches for natives of the western Alaska region. Some of these closures stem from the inability of fisheries managers to differentiate a late run that is of normal size from a small run that is returning at a normal date. One way that managers can differentiate these two scenarios is with stock composition estimates facilitated by panels of high-throughput SNPs. Specifically, stock composition estimates from mixed-stock fisheries and test fisheries on the high seas can be used to monitor the contribution of each stock in real time, helping to inform the need for fisheries closures and generally improving fisheries management (Seeb et al. 2000; Smith et al. 2005; Dann et al. 2013). Despite this potential utility, tools for genetic stock identification in marine waters of western Alaska have been severely hampered by lack of genetic divergence among regions (Templin et al. 2011). Our data provide the first evidence that assignment to region of origin is feasible in western Alaska despite low levels of divergence. Although it is not currently possible to screen 10 000 loci on thousands of individuals, a subset of our RAD loci that show high levels of divergence can be used to construct a high-throughput SNP panel to differentiate stocks in this region (c.f., Ackerman et al. 2011).

This high-throughput SNP panel could also be used to investigate the migration and distribution patterns of Chinook salmon on the high seas (e.g., Murphy et al. 2009; Larson et al. 2013). Patterns of productivity in the marine environment are thought to be a major cause of the fluctuations in abundance observed in Chinook salmon from western Alaska (Farley et al. 2005). Despite this variability, most stock assessment models assume a constant marine mortality rate across all stocks. The ability to monitor stock-specific abundance on the high seas could provide important information for stock assessment models which is currently unavailable. Additionally, stock composition estimates could be used to monitor the impact of Chinook salmon interception in the Bering Sea pollock fishery; this fishery has captured as many as 100 000 Chinook salmon in a single year (Gisclair 2009). In summary, our results represent the first step toward a panel of high-throughput SNPs that can be used to conduct genetic stock identification and improve stock-specific management in the western Alaska region.

### Applicability to other study systems

Our study demonstrates the utility of genomic data when attempting to differentiate closely related populations and estimate demographic parameters. The methods we employed will be especially applicable in marine species, which are often characterized by low genetic differentiation and large population sizes (Waples 1998; Nielsen and Kenchington 2001). For example, individual-based analyses with thousands of markers can provide extremely accurate estimates of individual genetic variation. Additionally, this method can shed light on patterns of connectivity by identifying migrants and admixture within populations.

Estimates of *N*_e_ in large marine populations can also be improved using approaches similar to ours (e.g., Gruenthal et al. 2014). Dense linkage maps have already been developed for many marine species including cod (Hubert et al. 2010), flounder (Castano-Sanchez et al. 2010), and shrimp (Du et al. 2010). By combining these linkage maps with genomic data, it may be possible to accurately estimate *N*_e_ and *N*_e_/*N* in many economically important marine species. These estimates can provide important insights into the adaptive potential of marine populations and can be used to inform management (Hare et al. 2011).

## Summary

Our results demonstrated fine-scale structure between regions in western Alaska. This structure allowed us to assign fish back to their region of origin with greater than 90% accuracy, representing a significant improvement over past studies. We also estimated *N*_e_ for each population using a novel method for nonmodel organisms. Estimates were generally large and provided some evidence that metapopulation dynamics influence demography in this region. Investigation of loci and genomic regions under putative selection found three potential regions of adaptive divergence. The methods described in our study will be particularly applicable to marine species or any species where large population size and shallow structure are common.

## Data archiving statement

Data for this study are available from the Dryad Digital Repository: http://dx.doi.org/10.5061/dryad.rs4v1. Illumina RAD-tag sequences have been submitted to the NCBI SRA database (accession numbers available upon request).

## Supporting Information

Figure S1 Histograms of locus-specific overall FST and pairwise FST for each population comparison.

Table S1 Summary statistics for all 10 944 loci.

Table S2 BLAST results for all 10 944 loci.

Table S3 Assignment probabilites to the three most likely populations for each individual and each SNP panel tested.
