# Supplementary material for: Genotyping by sequencing resolves shallow population structure to inform conservation of Chinook salmon (Oncorhynchus tshawytscha)
Source: Evol Appl. 2014 Jan 2;7(3):355–69. doi: 10.1111/eva.12128 (PMC3962296; doi:10.1111/eva.12128)
Supplement: Figure S1 — Histograms of locus-specific overall FST and pairwise FST for each population comparison. [file eva0007-0355-sd1.pdf]

Fig. S1. Histograms of locus-specific overall FST and pairwise FST for each population comparison. Population abbreviations are in Table S1.

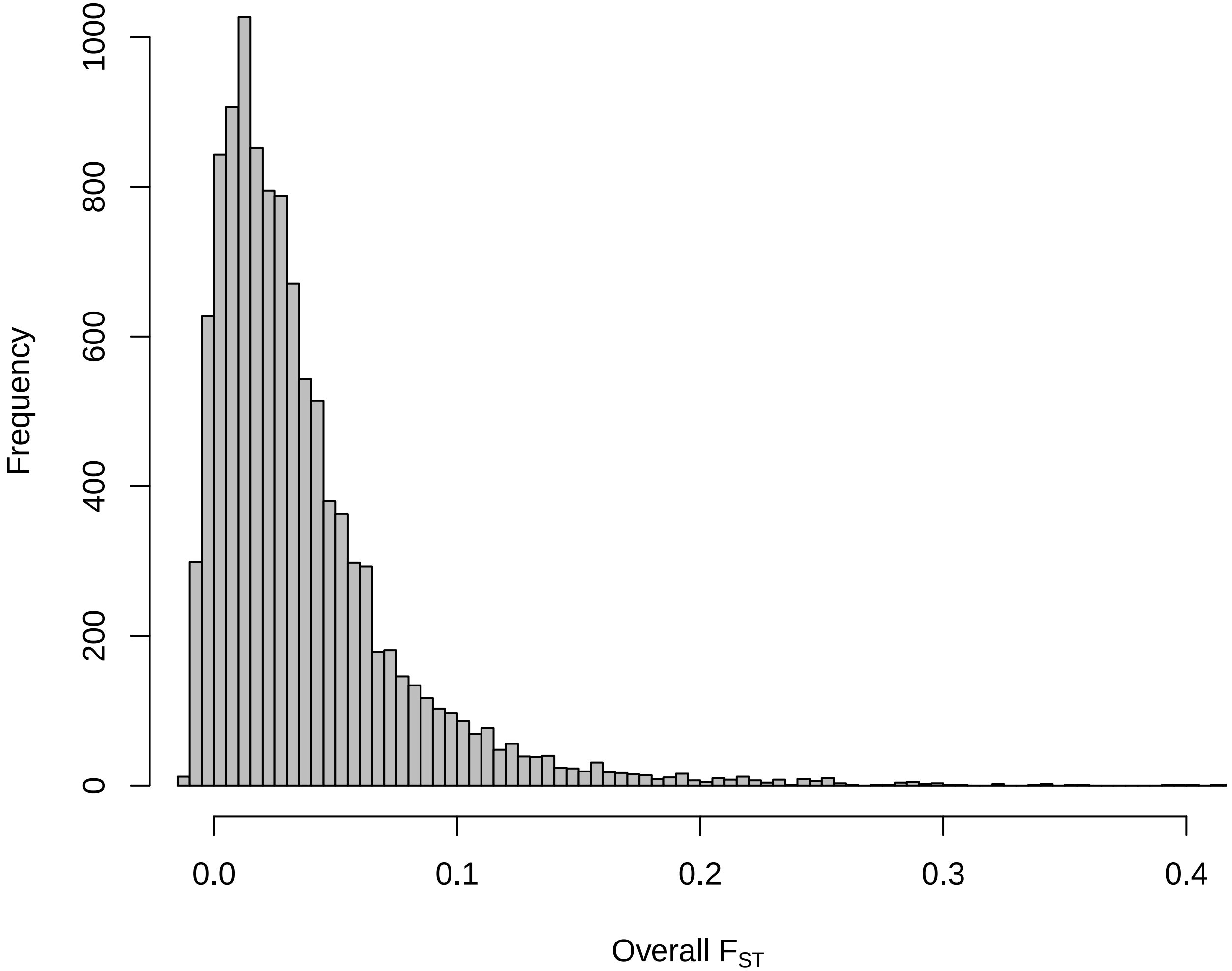

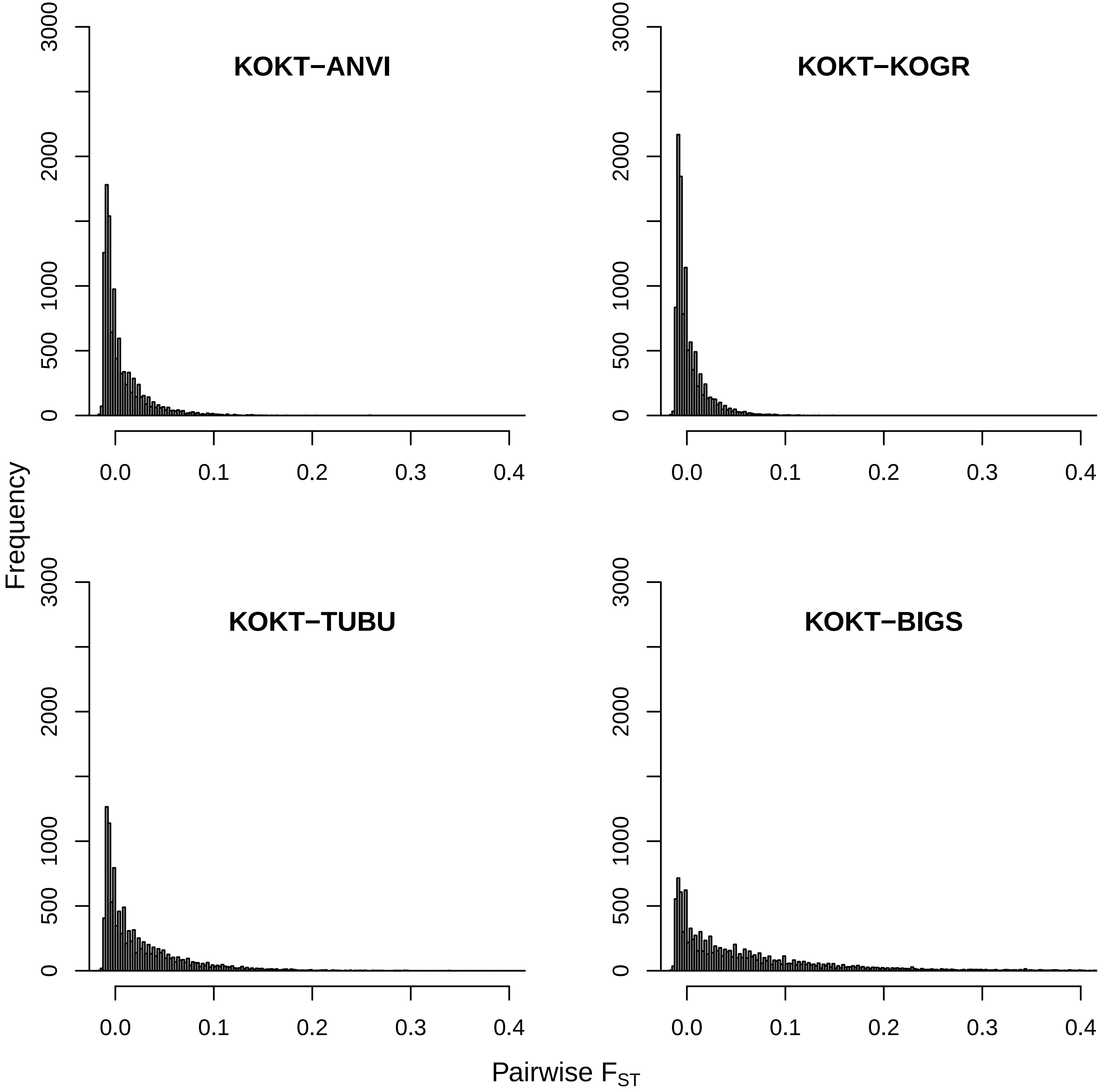
KOKT−ANVI
KOKT−KOGR
KOKT−TUBU
KOKT−BIGS
Frequency
0
500
1000
2000
3000
0.0
0.1
0.2
0.3
0.4
Pairwise F_{ST}

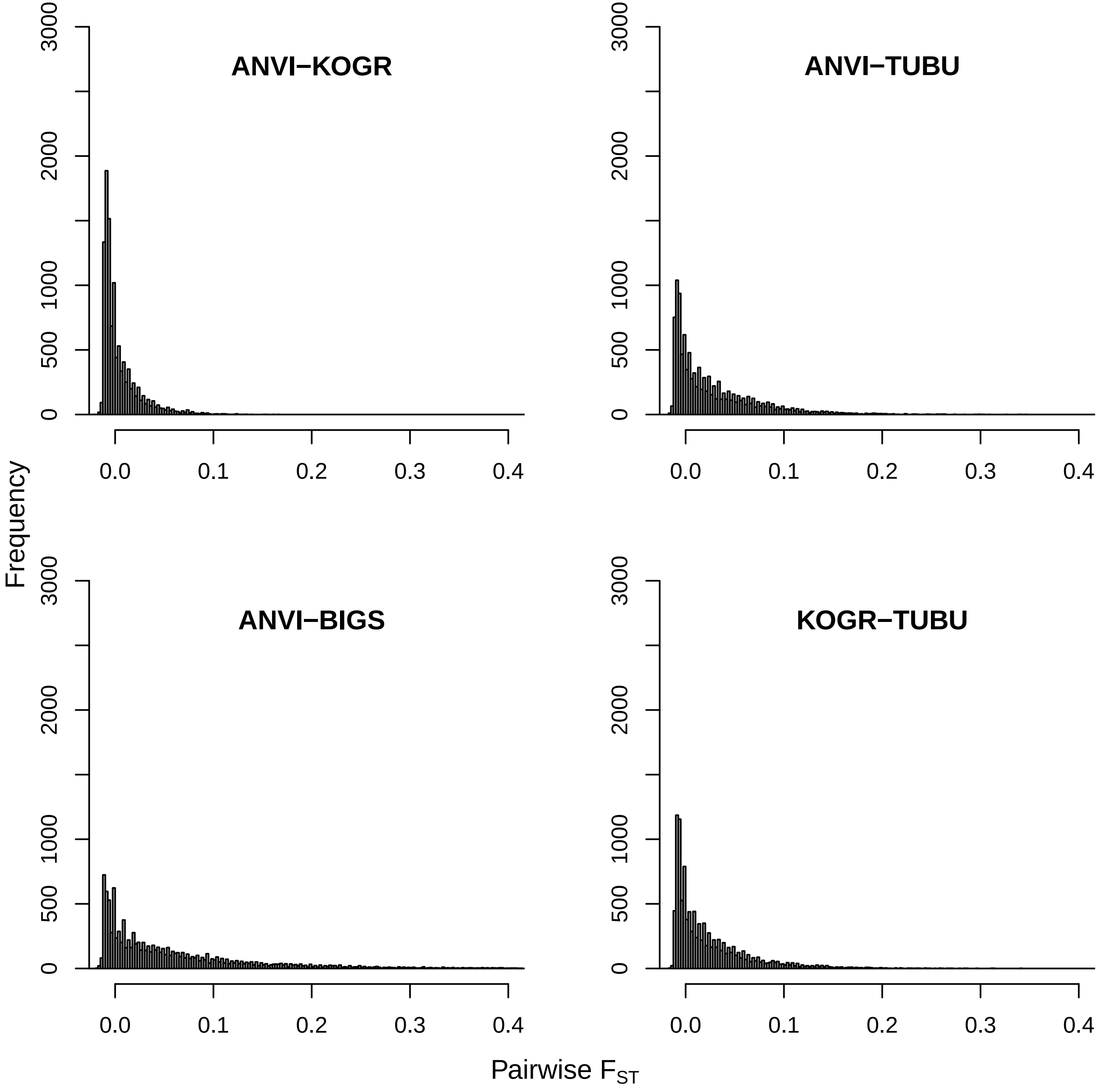

ANVI−KOGR
ANVI−TUBU
ANVI−BIGS
KOGR−TUBU
Frequency
0
500
1000
2000
3000
0.0
0.1
0.2
0.3
0.4
Pairwise F_{ST}

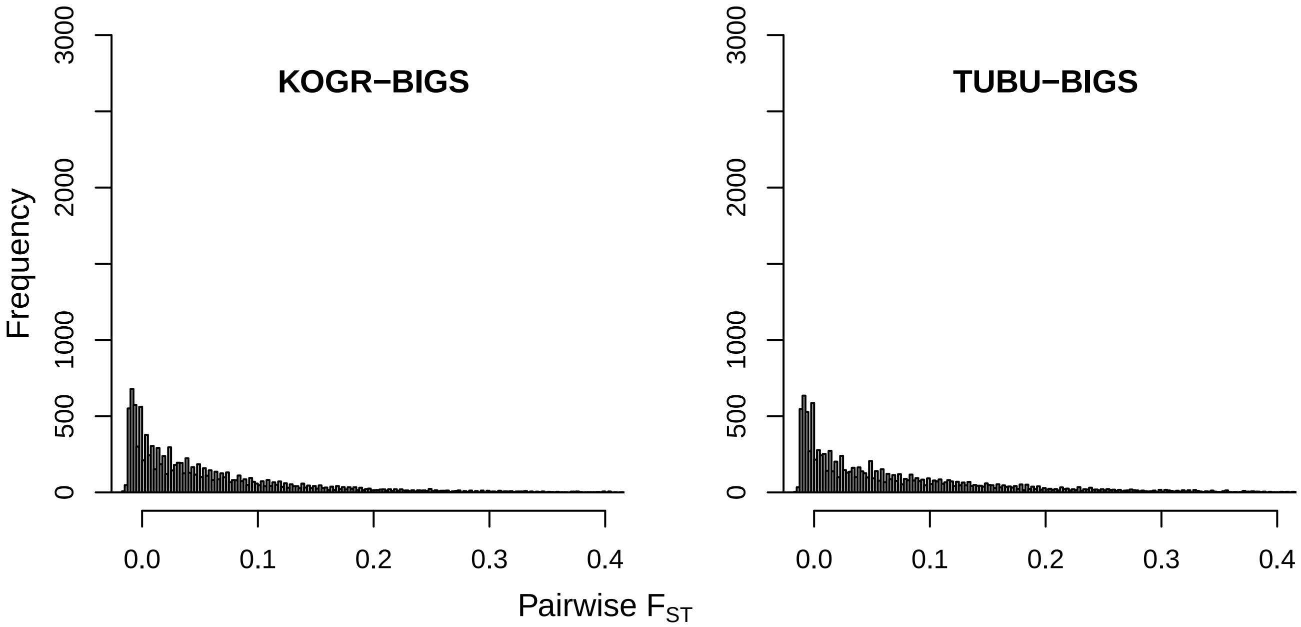
KOGR−BIGS
TUBU−BIGS
Frequency
0
500
1000
2000
3000
0.0
0.1
0.2
0.3
0.4
Pairwise F_{ST}
